# Machine learning model for detecting masked hypertension in young adults

**DOI:** 10.3389/fphys.2025.1684693

**Published:** 2025-11-17

**Authors:** Brendyn Miller, Samuel J. Coeyman, Annemarie Wentzel, Carina M. C. Mels, William J. Richardson

**Affiliations:** 1 Institute for Regenerative Medicine, Wake Forest University, Winston-Salem, NC, United States; 2 Ralph E. Martin Department of Chemical Engineering, University of Arkansas, Fayetteville, AR, United States; 3 Hypertension in Africa Research Team, North-West University, Potchefstroom, South Africa; 4 MRC Research Unit for Hypertension and Cardiovascular Disease, North-West University, Potchefstroom, South Africa

**Keywords:** machine learning, masked hypertension, African-PREDICT, cardiovascular disease, predictive modeling

## Abstract

**Introduction:**

Cardiovascular disease (CVD) remains the leading global cause of mortality, with hypertension (HT) being a significant contributor, responsible for 56% of CVD-related deaths. Masked hypertension (MHT), a condition where patients exhibit normotensive blood pressure (BP) in clinical settings but elevated BP in out-of-clinic measurements, poses an elevated risk for cardiovascular complications and often goes undiagnosed. Current diagnostic methods, such as ambulatory BP monitoring (ABPM) and home BP monitoring (HBPM), have limitations in feasibility and accessibility.

**Methods:**

This study aimed to address these challenges by leveraging machine learning (ML) models to predict MHT based on clinical data from a single outpatient visit. Utilizing a dataset from the African-PREDICT study, which included comprehensive clinical, biomarker, body composition, and physical activity data from a young, healthy cohort (aged 20–30 years) in South Africa, we developed a predictive framework for MHT detection.

**Results:**

The ML models demonstrated the potential to enhance early identification and treatment of MHT, reducing reliance on resource-intensive methods like ABPM. Specifically, we found that utilizing a Least Absolute Shrinkage and Selection Operator (LASSO) feature selection method with an extreme gradient boosting model had an accuracy of 0.83 and a ROC AUC score of 0.86 while relying predominantly on four features: systolic blood pressure, body weight, left ventricular mass at systole, and circulating levels of dehydroepiandrosterone sulfate.

**Discussion:**

This approach could enable targeted interventions, particularly in resource-limited settings, thereby mitigating the progression of MHT and its associated risks. These findings underscore the importance of integrating advanced computational techniques into clinical practice to address global health challenges.

## 1 Introduction

Cardiovascular disease (CVD) is the leading cause of death globally, claiming approximately 17.9 million lives annually (World Health Organization, 2023; Mathers et al., 2009). Hypertension (HT) is one of the strongest risk factors for CVD and is associated with coronary disease, left ventricular hypertrophy, valvular heart disease, cardiac arrhythmias, cerebral stroke, and renal failure (Kjeldsen, 2018). HT accounts for approximately 56% of all CVD-related deaths (10 million) and is incredibly prevalent, affecting an estimated 1.3 billion people worldwide (World Heart Federation, 2022). This number has been increasing and is expected to reach 1.56 billion deaths annually by the year 2025 due to multiple factors, including population aging, increased prevalence of chronic kidney disease (CKD), diabetes mellitus, and obesity, suboptimal clinical treatments, and poor adherence to treatment plans (Kearney et al., 2005; Hunter et al., 2021). HT is defined clinically as a blood pressure (BP) of 140/90 mmHg or higher and can be prevented or managed through lifestyle and pharmacological interventions (Messerli et al., 2007; Nguyen et al., 2010).

While HT can be managed, it often remains untreated as several population studies have found 12.7%–37.3% of all cases of HT were not diagnosed clinically (Huguet et al., 2021; Shukla et al., 2015; Essa et al., 2022). This is due in part to a subset of these patients (10% of the general population) having normotensive BP measurements within the clinic while their out-of-clinic BP as measured by ambulatory BP monitoring is elevated to the point of being considered hypertensive (Pickering et al., 2007). This condition has been classified as masked hypertension (MHT) and has been shown to have equal, if not increased, risk for adverse cardiovascular morbidity due to the lack of any clinical diagnosis and corresponding clinical intervention (Stergiou et al., 2014; Thakkar et al., 2020; Bobrie et al., 2008). Furthermore, this condition has been associated with increased organ damage, altered cardiovascular dysfunction and structural changes, and higher incidence of cardiovascular and cerebral events (including stroke and cognitive decline) (Bobrie et al., 2008; Trachsel et al., 2015; Fujiwara et al., 2018).

One recent meta-analysis has suggested that nearly one in three patients who have normotensive office blood pressure measurements have MHT. While this condition is more commonly present in older populations, MHT has even been identified in young and apparently healthy populations in the absence of clinically relevant risk factors (such as dyslipidemia, hyperglycemia, obesity, etc.) (Bobrie et al., 2008). Other studies have also reported similar findings, with one study reporting that approximately 11% of children under the age of 15 had MHT (Stergiou G. S. et al., 2005) and another study reported the prevalence of MHT in young to middle-aged adults (44 ± 19 years of age) to be 23% (Bendov et al., 2005). Other studies found MHT present in populations that appeared to be in peak physical condition, such as endurance runners and professional soccer players (Trachsel et al., 2015; Berge et al., 2013). These studies demonstrate the need to monitor the out-of-office BP of the general population to diagnose and treat MHT in a timely and effective manner. The most common methods for detecting MHT are ambulatory BP monitoring (ABPM) and home BP monitoring (HBPM), however both methods come with significant drawbacks (Hermida et al., 2015; Anstey et al., 2018). HBPM, while convenient and easy to obtain, has been shown to have a reduced ability to detect MHT when compared to ABPM and research published by Stergiou et al., suggests that HBPM should only be used in conjunction with ABPM to detect MHT (Stergiou G. et al., 2005). ABPM, on the other hand, has been shown to successfully detect MHT with a high degree of accuracy; however, it requires the use of cumbersome equipment that may not be available to certain population groups, particularly in children or in populations in low-and-middle income countries (Flynn et al., 2022; Shimbo et al., 2015; Stergiou et al., 2021; Abdalla, 2017). Thus, the feasibility of ABPM for population-level detection of MHT is unknown (Abdalla, 2017). One potential alternative would be to develop a method for assessing risk for MHT based on clinical measurements obtained from a single outpatient visit. This could serve as a preliminary screening method that would allow the patients most at risk for MHT to be identified while reducing the need for all patients to undergo ABPM. Patients that are classified as being at risk for MHT could then undergo ABPM to confirm the presence of MHT and the need for further medical and lifestyle intervention to prevent the advent of CVD.

Several studies have recently developed machine learning (ML) models to predict adverse cardiovascular events such as coronary heart disease, heart failure, and stroke that have shown potential to assist clinicians in early disease detection and diagnosis (Krittanawong et al., 2020; Sevakula et al., 2020). These models evaluate clinical features to determine which patients are most at risk for these events using a combination of statistical methods and computational algorithms that can be automatically fine-tuned to these specific applications based on the input data. Researchers have found that these data-driven models can outperform traditional models in applications involving a multitude of different variables due to their inherent ability to capture the non-linear relationships between these features and the variable that is being predicted (Sevakula et al., 2020; Motwani et al., 2016; Churpek et al., 2016). ML models are also useful for establishing a predictive model in which an experimentally validated model is not readily available. The goal of this study was to develop an ML model to detect patients with MHT in the hopes of improving MHT detection, diagnosis, and treatment in a young and relatively healthy population.

## 2 Materials and methods

### 2.1 Patient data acquisition

The dataset used in this model was derived from the African-PREDICT study, which aims at preemptively identifying cardiovascular disease in young adults from South Africa (Mokwatsi, 2022). The African-PREDICT study design and specific research methods used to collect the data have been described previously (Schutte et al., 2019). In brief, a total of 1202 black (N = 606) and white (N = 596) young men and women (aged 20–30 years) in South Africa were screened to be healthy and clinically normotensive and without complicating factors such as pregnancy or previous self-reported diagnosis of any chronic diseases. Different clinical measures relevant to hypertension were collected from each patient. Each of the measured features could be broadly categorized into the following groups:

1. Questionnaire data (e.g., medical history, social status, diet, psychosocial profile)
2. Biomarker data (e.g., lipids, glucose, multiplex cytokines, RAS-Fingerprint, adipokines, oxidative stress, nitric oxide and coagulation markers, urinary sodium, metabolomics, proteomics)
3. Body composition data (physical measurements)
4. Physical activity (vigorous, moderate, and sedentary activity levels)
5. BP (office, 24-h cuff monitoring, central, reactivity)
6. Target organ damage (arterial stiffness, carotid wall thickness, electrocardiography, echocardiography, retinal microvasculature, renal function).

Each data type was obtained at a single time-point upon patient enrollment in the study, with no follow-up data included in the present analysis. Office BP was measured 4 times, twice on both arms; 24-h BP was obtained over a single 24-h period; biomarker samples were obtained once, but appropriate duplicate/triplicate readings were used for biochemical panel measurements. All measurements were conducted in accordance with current gold standard methodologies and were carried out by trained research nurses, postgraduate students, and academic staff. The necessary ethical clearance was obtained from the Health Research Ethics Committee of North-West University (ethics number: NWU-00001–12-A1), with all participants providing written informed consent prior to data collection; the study is registered on ClinicalTrials.gov (Identifier: NCT03292094).

### 2.2 Machine learning model overview

**FIGURE 1:**
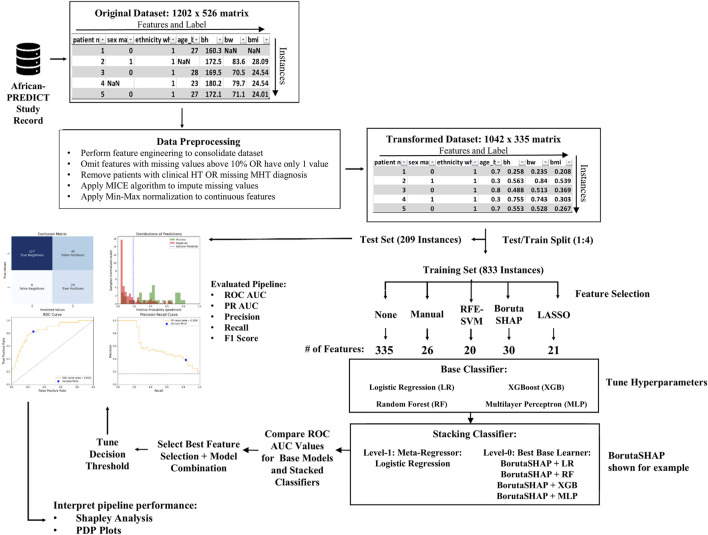
Overview of pipeline development process. The original dataset was imported as a 1202 × 526 matrix. It underwent preprocessing that consisted of feature engineering, data cleaning, imputation, and scaling to make a transformed dataset that was a 1042 × 335 matrix. The data was split into testing and training sets using a 1:4 split ratio. Five feature selection methods were evaluated on four different base ML models and a fifth ensemble ML model, resulting in 25 different pipelines (feature selection algorithm + ML model). The best performing pipelines were evaluated and SHAP analysis was used in conjunction with PDP plots to interpret the pipeline predictions.

Figure 1 depicts the overall process implemented during machine learning model development. The original African-PREDICT dataset contains health records with 526 different features from a cohort of 1,202 de-identified patients. This dataset was preprocessed prior to modeling and then split into a training set for constructing the ML model and a testing set for validating the model. One of five different feature selection strategies was then applied to the training set to identify the relevant features in the dataset and eliminate the unnecessary features. The relevant features were used to train the model using four different classification models as well as a stacked model assembled with all the base models combined. This resulted in 25 different pipelines in total (5 feature selection algorithms x five different ML classification models).

The overall goal was to correctly classify patients with MHT. In this cohort, MHT was defined as the absence of hypertension in clinical measurements (i.e., clinic systolic blood pressure <140 mmHg and diastolic blood pressure <90 mmHg based on European Society of Hypertension guidelines) combined with high blood pressure in 24-h ambulatory cuff monitoring (i.e., systolic blood pressure ≥130 mmHg and/or diastolic blood pressure ≥80 mmHg). These ambulatory blood pressure measurements were acquired by fitting a cuff to participants’ non-dominant arms, and instructions given to ensure successful inflations. The cuff was programmed for readings every 30 min during the day and every 60 min during the night. Successful blood pressure readings were averaged across the measurement period and then compared to the systolic and diastolic pressure thresholds.

The model performance for each feature selection strategy was compared using the area under their receiver operating characteristic curve (ROC AUC). The classifier with the highest ROC AUC was then tuned, and the results of the final model were reported. Shapley Additive explanations (SHAP) values were used to investigate the individual contributions of each feature to the model predictions, and partial dependence plots (PDPs) were created to assess the relationship between each feature and the predicted outcome (Gramegna and Giudici, 2022).

### 2.3 Data preprocessing

The first step in building the ML model was to load the de-identified patient data and store it as a data frame in Python (version 3.12.2) using an open-source integrated development environment (IDE) (Visual Studio Code V1.93). The dataset was first cleaned by dropping any patient who was clinically diagnosed with hypertension, any patient who was missing more than 10% of feature measurements, and any feature that was missing in more than 10% of patients. Any features that contained only one unique value in the dataset were also dropped.

After cleaning the data, the next preprocessing step was to drop irrelevant features and perform feature engineering to reduce the number of features in the dataset. The purpose of this step was two-fold: first, reducing the number of features in the dataset by removing unnecessary features helps to improve model performance and reduce computational cost (Guyon and De, 2003), and second, this step helps to improve the overall interpretability of the model. Several features in the original dataset were not considered relevant to the model based on the intended use case for the model. These features could be broadly classified as ambulatory blood pressure measurements and certain questionnaire features. Next, we performed a literature review of known risk factors for HT (Franklin et al., 2015; Franklin et al., 2016; Booth et al., 2016; du Toit et al., 2023). Of these, several categorical features were identified that could be determined from numerical features in the dataset. Given that these categorical features could potentially help improve the performance of certain ML models and potentially serve as important predictors of MHT, these features were created using the criteria outlined in Table 1 and were included as additional features in the data frame.

**TABLE 1:** List of engineered categorical features and corresponding definitions based on previous analyses (Franklin et al., 2015; Franklin et al., 2016; Booth et al., 2016; du Toit et al., 2023).

Feature Name	Variable type	Classification	Definition
Prehypertensive	Categorical	0-healthy, 1-diseased	140mmHg > SBP >119mmHg OR 90mmHg > DBP >79mmHg
Overweight	Categorical	0-healthy, 1-diseased	BMI >25
Left Ventricular Hypertrophy	Categorical	0-healthy, 1-diseased	Left Ventricular Mass Index >115 g/m^2^ AND Sex is Male Left Ventricular Mass Index >95 g/m^2^ AND Sex is Female
Physically Inactive	Categorical	0-healthy, 1-diseased	<600 METs for moderate and/or vigorous activity
Smoker	Categorical	0-healthy, 1-diseased	Self-identified smoker AND continine ≥11 ng/mL
Excessive Alcohol Use	Categorical	0-healthy, 1-diseased	Self-identified alcohol consumption AND GGT ≥49 U/L
Dyslipidemic	Categorical	0-healthy, 1-diseased	LDL >3.4 mmol/L

Once the dataset had been cleaned and feature engineering had been conducted, there was a total of 335 features across 1042 patients. Next, any remaining missing numerical values in the dataset were imputed using the Multivariate Imputation by Chained Equations (MICE) algorithm (van Buuren and Groothuis-Oudshoorn, 2011). The MICE algorithm assumes that the missing values are missing at random without any underlying relationship between the instances where features are missing and a detailed explanation of the algorithm has been described by Azur et al., (Azur et al., 2011). For this work, MICE imputation was implemented using the iterative imputer class in scikit-learn. More details of how this algorithm was implemented in the scikit-learn library are described elsewhere (van Buuren and Groothuis-Oudshoorn, 2011; Pedregosa, 2011). For the categorical features, missing values were filled in using the mode of the entire column.

After imputing missing values in the dataset, the final step in the data preprocessing stage was to scale the continuous features to increase model efficiency and prevent feature bias from occurring in the ML algorithms that weigh feature importance based on Euclidean distance measures. This is a crucial step for those ML algorithms, as features of high magnitude can be biased toward higher weights than features of lower magnitude (Jadhav et al., 2019). Scaling the dataset was implemented using a MinMaxScaler class from the scikit-learn.preprocessing library (scikit-learn v. 1:3:0) (Pedregosa, 2011).

### 2.4 Feature selection

The next step in ML model development was to perform feature selection, which is a crucial to preparing a dataset for training a ML model (Kumar, 2014). This phase was especially important in the present study because of a high participant-to-variable ratio (1042:335). Feature selection enabled a drastic reduction in the number of features fed into the subsequent ML models, which can improve training performance. For this work, a total of five different feature selection methods were explored:

1. RFE-SVM–a wrapper feature selection method known as recursive feature elimination which utilizes a support vector machine classifier
2. BorutaSHAP–a wrapper feature selection method which utilizes the Boruta algorithm wrapped around an extreme gradient boosted classifier (XGB) to select features based on their SHapley Additive exPlanation (SHAP) values
3. LASSO - an embedded feature selection method that uses an L1 regularization technique to eliminate unimportant features by shrinking the coefficients of these features to zero and effectively removing them from the model, known as Least Absolute Shrinkage and Selection Operator regression.
4. Manual–features based on relevant predictors reported in scientific literature
5. None–all features included

RFE-SVM is a popular wrapper feature selection method that has been used in multiple previous studies to select important features using the backward feature elimination algorithm in conjunction with a SVM linear classifier (Samb et al., 2012). SVM classifiers are a class of generalized linear classifiers that operate under the guiding principle of simultaneously minimizing classification error and maximizing the geometric margin by identifying the hyperplane that maximizes the Euclidian distance between the plane and the dataset features in a multidimensional space (Brereton and Lloyd, 2010). For this study, the Recursive Feature Elimination was implemented using the RFE class in the scikit-learn.feature_selection library.

The second automatic feature selection technique that was evaluated for this study was the Boruta-SHAP technique. Briefly, the Boruta method determines feature importance by comparing the relevance of real features in the dataset to randomized copies of the features. A more detailed overview of the Boruta algorithm has been described previously by Kursa et al., (Kursa et al., 2010). For this study, the standard Boruta-SHAP algorithm was slightly modified to utilize SHAP values as the metric for importance scoring. This metric was selected based on previous research which has evaluated different automatic feature selection techniques and found that feature selection techniques based on SHAP values are more stable (less lightly to alter selected features for different permutations of the training data) and can improve overall model performance compared to other automatic feature selection techniques (Gramegna and Giudici, 2022; Ma and Huang, 2008). The Boruta-SHAP feature selection algorithm was implemented in python using the BorutaSHAP function BorutaSHAP library (BorutaSHAP v. 1.0.17) (Keany, 2020). A Random Forest Classifier class from the scikit-learn.ensemble library was fit to a training dataset containing all the features and implemented as the wrapper for the BortuaSHAP instance.

The third and final automatic feature selection method that was assessed in this study was LASSO regression. At a high level, LASSO regression performs feature selection by shrinking the coefficient of unimportant features in the regression model towards zero through the introduction of an L1 regularization penalty term (Ranstam and Cook, 2018). LASSO regression feature selection was implemented using the LassoCV class in the scikit-learn.linear_model library.

For the manual feature selection, a literature review was conducted to determine which features in the original dataset were correlated with the incidence of MHT. Features from our dataset that were found to be relevant to MHT are shown in Table 2.

**TABLE 2:** Manual selected features based on literature reviews.

Patient information	Blood lipid levels	Personal/Family medical history
Sex (Bobrie et al., 2008; Franklin et al., 2015; Franklin et al., 2016; Booth et al., 2016; Longo et al., 2005; Hung et al., 2021; Barochiner et al., 2013; Mallion et al., 2006; Hänninen et al., 2011) Ethnicity (Franklin et al., 2015; Franklin et al., 2016; Booth et al., 2016) Age (Bobrie et al., 2008; Franklin et al., 2015; Franklin et al., 2016; Booth et al., 2016; Longo et al., 2005; Hung et al., 2021; Hänninen et al., 2011)	Glucose (Franklin et al., 2016; Hung et al., 2021) Triglycerides (Franklin et al., 2016; Hung et al., 2021) LDL (Franklin et al., 2016; Hung et al., 2021) HDL (Franklin et al., 2016; Hung et al., 2021) Total Cholesterol (Franklin et al., 2016; Hung et al., 2021) MCP-1 (Thompson et al., 2016) CRP (Thompson et al., 2016)	Prehypertension (Bobrie et al., 2008; Franklin et al., 2015; Booth et al., 2016) Obesity (Longo et al., 2005; Hung et al., 2021) Stroke (Franklin et al., 2015; Longo et al., 2005) Diabetes Mellitus (Franklin et al., 2015; Franklin et al., 2016; Booth et al., 2016; Longo et al., 2005; Hänninen et al., 2011)

Clinical Measurements	Lifestyle Factors	Echocardiogram Measures
Systolic Blood Pressure (Bobrie et al., 2008; Franklin et al., 2016; Booth et al., 2016; Hung et al., 2021; Barochiner et al., 2013; Mallion et al., 2006; Hänninen et al., 2011) Diastolic Blood Pressure (Franklin et al., 2016; Booth et al., 2016; Hung et al., 2021; Hänninen et al., 2011) Mean Arterial Pressure (Hung et al., 2021) Pulse Pressure (Hung et al., 2021) Clinic Heart Rate (Hänninen et al., 2011) Pulse Wave Velocity (Thompson et al., 2016)	Smoking (Bobrie et al., 2008; Franklin et al., 2015; Franklin et al., 2016; Booth et al., 2016; Longo et al., 2005; Hänninen et al., 2011) Alcohol Consumption (Bobrie et al., 2008; Franklin et al., 2015; Longo et al., 2005; Barochiner et al., 2013; Mallion et al., 2006; Hänninen et al., 2011) Physical Activity Level (Franklin et al., 2015; Longo et al., 2005)	Left Ventricular Mass (Franklin et al., 2015; Franklin et al., 2016; Longo et al., 2005) Left Ventricular Hypertrophy (Franklin et al., 2015; Hänninen et al., 2011) Intima-media Carotid Thickness (Longo et al., 2005)

### 2.5 Machine learning models

The overall goal of this model was to detect patients that were at risk for MHT, so patient classification was the ML task chosen to be modeled using the African-PREDICT dataset. Several ML classifier algorithms were employed and evaluated for this purpose using the features from each feature selection strategy. These algorithms included:

1. Multivariate logistic regression (LR) classifier
2. Random forest (RF) classifier
3. Extreme Gradient Boosting (XGB) classifier
4. Artificial Neural Network (ANN) classifier
5. Stacking (STK) classifier

### 2.6 Hyperparameter & decision threshold tuning

A Bayesian Optimization grid search strategy was employed to evaluate a range of hyperparameters for each model (Table 3). Hyperparameters in a set were mapped to a corresponding score probability to create a probabilistic model that enabled the grid search to converge to the optimal hyperparameter values, rather than blindly testing each combination of values individually. A more detailed discussion of this algorithm is presented by Wu et al. (2019). Five-fold cross-validation was used for hyperparameter tuning and model training to help improve how the fitted values would generalize to a test dataset.

**TABLE 3:** List of ML models and their associated sets of evaluated hyperparameters.

ML model	Hyperparameters
LR	‘C': [0.1, 1, 10] ‘penalty’: ['l1′, 'l2'] ‘solver’: ['liblinear','saga'] ‘class_weight': [None,'balanced']
RF	‘n_estimators’: [100, 200, 300] ‘max_depth’: [None, 5, 10, 15] ‘min_samples_split’: [2, 5, 10] ‘min_samples_leaf’: [1, 2, 4] ‘max_features’: [None,'sqrt','log2'] ‘criterion’: ['gini’, 'entropy'] ‘class_weight': [None,'balanced']
XGB	‘n_estimators’: [100, 200, 300] ‘learning_rate’: [0.1, 0.05, 0.01] ‘max_depth’: [3, 5, 10] ‘subsample’: [0.5, 1.0, 'uniform'] ‘gamma': [0, 5.0] ‘colsample_bytree’: [0.8, 0.9, 1.0]
MLP	‘activation': ['relu','tanh'] ‘solver': ['sgd','adam'] ‘learning_rate': ['constant','adaptive'] ‘alpha': [0.0001, 0.001, 0.01]

The final step in constructing the overall pipelines was to tune the decision threshold value for classifying patients as “at risk” for MHT. For every model, the predicted MHT probability for each patient was calculated. This decision threshold was again cross-validated with a ten-fold repetitive split of the training data.

### 2.7 Evaluation metrics

This study evaluated several classification metrics commonly used to evaluate the performance of an ML model on an imbalanced dataset. These metrics include the accuracy, precision, recall (also known as sensitivity or true positive rate), specificity (equal to one minus the false positive rate), the F1 score, area under the receiver operator characteristic curve (ROC AUC), area under the precision-recall curve (PR AUC), and the odds ratio. Each of these metrics provides information on how well the model was able to identify patient outcomes based on the features in the dataset. ROC AUC is one of the most common evaluation metrics for binary classification problems and is constructed by plotting the true positive rate (TPR) vs. the false positive rate (FPR) for a range of decision thresholds between 0 and one and then calculating the area under the resulting curve.

### 2.8 Model interpretation

Model interpretability is a key factor when developing a ML model for clinical applications as end users, such as clinicians and other medical professionals, need to be able to understand how a prediction was made in order to confirm that the prediction is valid and aligns with their own medical knowledge and understanding. Thus, a method for helping model end users to make sense of the model is imperative when the final model involves more complex ML algorithms (Cinà, 2022). Two common methods used in ML model development include SHAP Values and Partial Dependence Plots (PDPs). SHAP values are used to access the contribution of a feature to a model’s predicted outcome for a particular instance is a concept rooted in game theory (Hart, 1989). In its original context, Shapley values were created as a means of evaluating a player’s contribution to a game’s outcome. It has since been applied in the field of ML as a model-agnostic means of interpreting how a model operates (Shih et al., 2022). In this case, each feature in the model is considered a player in the game and the model prediction is the outcome.

PDPs are a valuable tool for interpreting and explaining the behavior of ML models by providing insights into how the model’s predicted outcome changes as a function of a specific feature while holding other features constant. By isolating the effect of a single feature on the model’s predictions, PDPs highlight the relationship between that feature and the output feature in an intuitive and visual manner. These plots can then be used to identify trends, patterns, and potential nonlinearities that might not be evident from simple summary statistics or coefficients. PDPs can also facilitate the detection of interaction effects between variables, showcasing how their combined influence impacts the model’s output.

## 3 Results

### 3.1 Feature selection and relative importance of selected features

**FIGURE 2:**
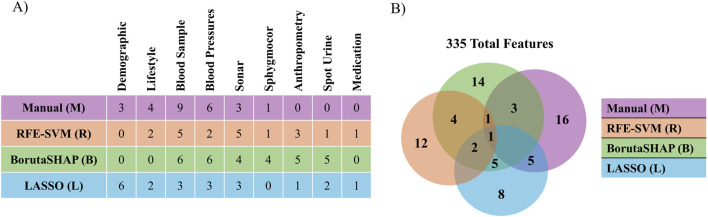
Comparison of features selected by each selection method. **(A)** The number of features selected by each method for each patient data category. **(B)** The number of features selected by and shared by each method.

Five different feature selection strategies were evaluated in this study: RFE-SVM, BorutaSHAP, LASSO, manual, and none (all features). The number of selected features for each feature selection strategy are reported in Figure 2A with common features found across the selection strategies shown in Figure 2B. Of all the feature selection strategies that were evaluated, the LASSO method produced the highest ROC AUC value using the XGB classifier, with a total of 21 features selected. Dyslipidemic, LV posterior wall thickness at diastole, phosphorus, prehypertensive, medication for alimentary tract and metabolism, socio-economic education, socio-economic household income, and socio-economic skill were unique to LASSO; body weight, chromium, blood pressure grades, c-peptide, and LV posterior wall thickness at systole were shared between LASSO and BorutaSHAP; participant age, alcohol consumption, ethnicity, obesity, and sex were shared between LASSO and the manual selections; LV mass at systole and dehydroepiandrosterone sulfate (DHEA-S) was shared between the LASSO, RFE-SVM and BorutaSHAP strategies; and systolic blood pressure was shared by all strategies. It is important to note that this selection includes a variety class of features from biochemical analyses, general questionnaire, and anthropometry measurements.

### 3.2 Comparison of classifiers across different feature selection strategies

**FIGURE 3:**
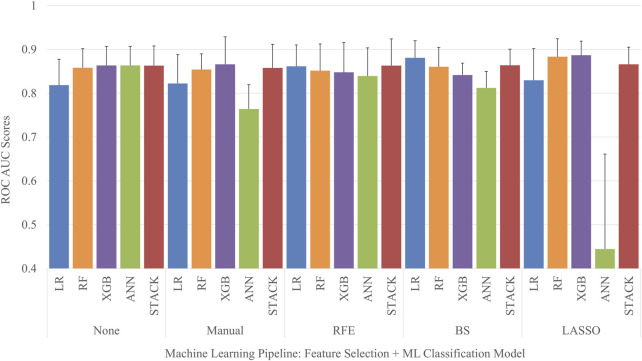
ROC AUC comparison of machine learning models.

Each permutation of feature selection strategy and machine learning algorithm was implemented to create a total of 25 different MHT classifier pipelines. Five-fold cross-validation was used to obtain the mean and standard deviation of the ROC AUC for each model, seen in Figure 3. The LASSO feature selection strategy combined with a XGB classifier obtained the highest average ROC AUC score out of the entire set of models when evaluated on the test set, with a ROC AUC of 0.89 ± 0.03. Conversely, the ANN classifier tuned using the features selected with LASSO obtained the lowest average ROC AUC score out of the entire set of models when evaluated on the test set, with a ROC AUC of 0.44 ± 0.22.

### 3.3 Evaluation of final model performance

**FIGURE 4:**
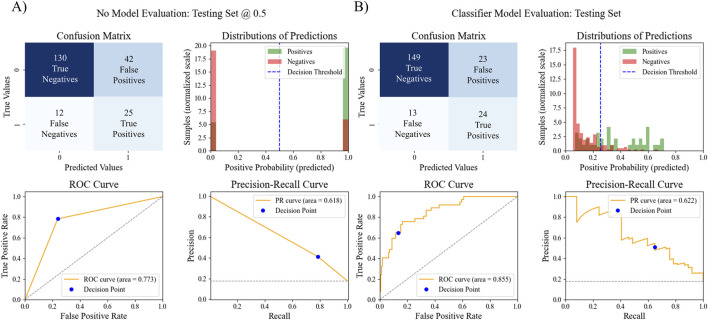
Comparison of MHT ML model performance vs. MHT binary model performance. The confusion matrix, distribution of predictions, ROC AUC, and PR AUC for **(A)** the simple binary classifier and **(B)** the best ML pipeline.

After examining all ROC AUC scores, the XGB classifier with LASSO feature selection strategy was chosen as the final pipeline for the MHT classifier due to it having the highest ROC AUC score. To assess the overall model performance, the pipeline’s accuracy, ROC AUC, PR AUC, F1 score, sensitivity, specificity, and odds ratio within the test set were calculated. We also compared these performance metrics to a binary prediction model previously proposed by Thompson et al. that classified MHT risk based on a simple cutoff for office systolic blood pressure greater than 120 mmHg (Thompson et al., 2016). The results of the comparison are outlined in Table 4 and Figure 4. The XGB classifier with LASSO features outperformed the binary classifier across all metrics evaluated, except for sensitivity where the binary had a slightly better sensitivity.

**TABLE 4:** ML model evaluation metrics vs. simple binary classifier evaluation metrics.

		Actual MHT		Accuracy	ROC AUC	PR AUC	F1 score	Sensitivity	Specificity	Odds ratio
		-	+							
Binary Prediction	-	130	12	0.742	0.719	0.557	0.481	0.676	0.756	6.45
	+	42	25							
ML Prediction	-	149	13	0.828	0.855	0.622	0.571	0.649	0.866	11.96
	+	23	24							

### 3.4 Model explanation

**FIGURE 5:**
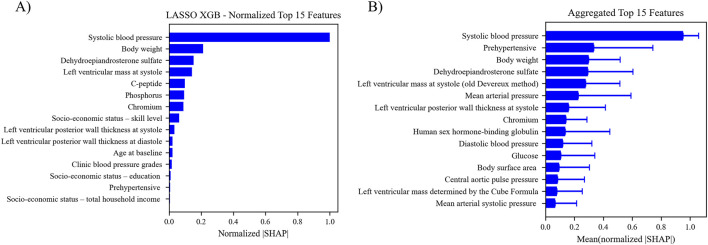
**(A)** Features ranking according to the mean absolute SHAP value. The absolute SHAP value of each feature for each instance was calculated and averaged to get the relative feature importance. **(B)** Aggregated feature ranking for features across the five top performing pipelines.

SHAP analysis was used to evaluate the overall contribution of each selected feature to the predictions made by the best five pipelines. SHAP values were calculated for each feature for each instance in the optimized dataset. The absolute value of each SHAP value for a particular feature across all instances were summed together, averaged, and normalized to indicate the importance of the feature relative to the other features in the optimized dataset. The normalized values were aggregated across the five pipelines with the highest test ROC AUC, and the top 15 features are shown in Figure 5. Plots of original and normalized values for each of the five best pipelines can be seen in supplemental figures, along with ‘bee swarm’ plots for each model. The top five features used in the best pipeline (LASSO XGB) were the same as the top five features across the aggregate of the five best pipelines: systolic BP, prehypertensive diagnosis, body weight, DHEA-S, and LV mass.

**FIGURE 6:**
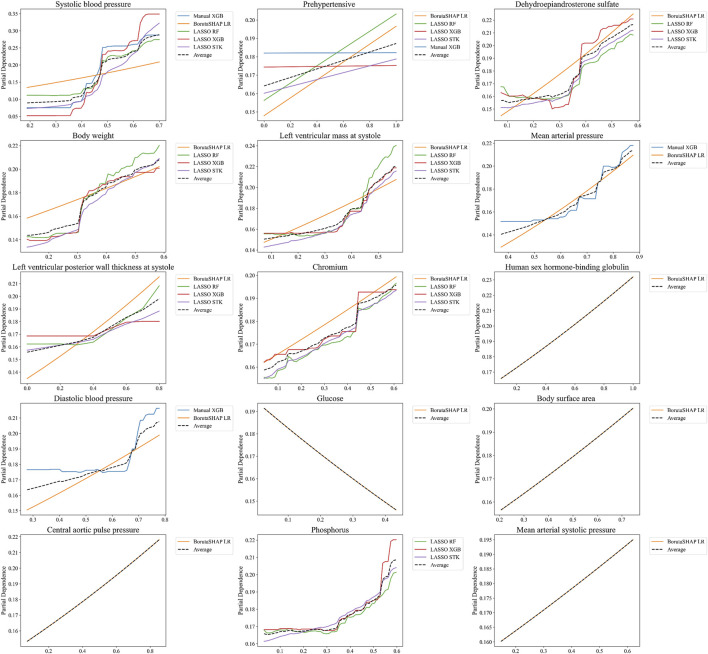
Partial Dependence Plots for features selected from the aggregated rankings. PDPs are shown for only the top 15 most important features determined by SHAP values in Figure 5, averaged for the five best ML pipelines. Note that not every one of these features was included in the top five pipelines, which is why some features only include 1, 2, or 4 PDP curves.

The relationship between the predicted outcome of the model using each of the top 15 LASSO XGB features, and the top 15 aggregated features were also assessed with PDPs (Figure 6). This analysis indicated that the best pipelines’ chances of classifying a patient with MHT increased in conjunction with elevated BPs, body weight, body surface area, LV mass/thickness, and several biochemical markers (DHEA-S, chromium, human sex hormone-binding globulin). Conversely, the risk of MHT decreased in conjunction with elevated glucose levels. Individual PDPs for each of the five best pipelines can be seen in the Supplementary Material.

## 4 Discussion

The aim of this study was to develop a machine learning pipeline that would be able to detect MHT in a seemingly young and healthy individual from the African-PREDICT cohort. To develop the pipeline, several popular feature selection methods and ML algorithms were evaluated. The results of this study found that the LASSO feature selection strategy in conjunction with a XGB classifier resulted in a ML model that had the highest ROC AUC score out of the set of 25 different pipelines that were constructed. The final ML pipeline was compared to a simple binary classifier that served as a “rule-of-thumb” for evaluating a patient’s risk for MHT in LMIC and was found to perform much better in each evaluation metric that was considered, except for sensitivity where the binary model had a slightly better performance (Thompson et al., 2016). SHAP analysis and PDP plots were also implemented to help interpret the contributions of different features to the model predictions. SHAP analysis revealed that the most important predictors of MHT for this ML model were related to systolic blood pressure and patient body weight. Individual patient analysis using this model could be used to identify which of these features need to be focused on and could help clinicians in implementing a clinical action plan. Overall, the results of this study show that an ML pipeline could be utilized to identify patients most at risk for MHT and highlight which features provide the greatest predictive value.

The final model’s performance was evaluated on the test set, resulting in an accuracy of 0.828, a ROC AUC score of 0.855, a PR AUC score of 0.622, a precision score of 0.511, a sensitivity score of 0.649, a specificity score of 0.866, and an F1 score of 0.571. These evaluation metrics are comparable to the reported evaluation metrics for other recently published ML models for MHT (Hung et al., 2021; Shih et al., 2022; Lip et al., 2021) or better (Bae et al., 2022; Meng et al., 2022). In a previous study that evaluated the African-PREDICT dataset, an office measurement of systolic blood pressure over 120 mmHg could be used as a cut-off value for classifying someone as at-risk for MHT (Thompson et al., 2016). Therefore, for comparison with this study, a simple binary model was created that classified patients as having MHT if they had an office systolic blood pressure of 120 mmHg. With just this single predictor alone, this basic classification model resulted in an accuracy of 0.742, a ROC AUC score of 0.719, a PR AUC score of 0.557, an F1 score of 0.481, a sensitivity score of 0.676, a specificity score of 0.756, and precision score of 0.373. While the simple binary model performed fairly well, the ML pipeline did perform better in all aspects except for sensitivity. The primary strength to the new pipeline, and the potential argument for clinical adoption of this model, is the number of individuals that were successfully identified as not having MHT. When comparing the ML model results with the binary systolic pressure cutoff model, the ML model was able to correctly classify 19 more individuals as true negatives, while the simple binary model incorrectly classified those individuals as false positives. That means 19 people might have been prescribed unnecessary treatment or follow-up monitoring based on the binary prediction, resulting in wasted financial costs, potential side-effects, etc.

Model interpretability is crucial for promoting clinical use because it provides the clinician and patient with insight into why the model makes a particular prediction one way or the other. In addition, model explanations help point to potential underlying physiological relationships that provide a deeper understanding of the pathology and possible interventional strategies. Using SHAP analysis, it was shown that the five most important features for the best ML pipeline (LASSO + XGB) were the same five most important on average across the five best ML pipelines. These features included systolic BP measured in the clinic, a prehypertensive designation (based on clinic BP measurements), body weight, circulating serum levels of DHEA-S, and LV mass at systole measured with echocardiography. These features are all noninvasive measurements that could be performed in any clinic. It is important to note that four of these five features (BPs, DHEA-S, and LV mass) have all been directly linked to physiological stress and sympathetic control, and the fifth feature (body weight) is often related to waist circumference which has also been linked to physiological stress (Grassi et al., 2018; Borovac et al., 2020; Maninger et al., 2009; Kühnel et al., 2023; Tryon et al., 2013). During acute mental stress, DHEA-S levels typically increase transiently, peaking immediately after stress exposure and then gradually returning to baseline within about an hour - however, long-term or chronic psychosocial stress is associated with reduced basal levels of DHEA-S and a diminished capacity to produce DHEA-S during acute stress, suggesting an impaired adrenal response and potential link to adverse health outcomes and accelerated aging (Lennartsson et al., 2013). SBP increases during physiological stress due to sympathetic nervous system activation, causing vasoconstriction and increased cardiac output. The magnitude of SBP reactivity to stress varies among individuals and can be influenced by factors such as sex and lifestyle. For example, in women, higher DHEA-S levels have been linked to greater blood pressure reactivity to stress (Hirokawa et al., 2016). Chronic stress-induced hemodynamic load can also promote left ventricular hypertrophy, reflecting the cumulative impact of stress on cardiac structure (Wentzel et al., 2025). The fact that these factors played large roles in our ML model supports a connection between MHT and a possible pathological mechanism related to underlying stress–a relationship worth testing more directly in future studies. Further, interventions aimed at mitigating stress control could be a route to reduce MHT risk even without pharmacological interventions. Of course, it is important to note that SHAP values and PDPs indicate feature importance in modeling predictions but do not necessarily imply causal relationships.

There are several limitations to this study that are worth highlighting. First, the number of participants in the dataset was relatively small for the number of features that were evaluated, and it is likely that the model performance would increase given a larger set of training data. A broader population would especially improve the model’s predictive capabilities for more focused demographic subgroups based on sex, race, age, etc. Secondly, the model was not validated on a test set from an external cohort; thus, the general utility of this model has not been tested. Thirdly, our analyses are cross-sectional, and temporal ordering cannot be established. The reported findings should not be interpreted as causal and should be confirmed in future prospective analysis. Still, the ML approach enables efficient pattern discovery by identifying multivariate and nonlinear associations between clinical variables and outcomes, which conventional statistics may miss. This enables discovery of novel risk markers or biomarker combinations relevant to disease prediction or prognosis–even when cross sectional data is used. In application, this ML pipeline can be integrated into electronic health records or clinical decision support systems to provide physicians with data-driven risk assessments or intervention recommendations for conditions influenced by vascular and endocrine profiles. For example, an algorithm predicting elevated cardiometabolic risk based on a cross-sectional panel (including DHEA-S, blood pressure, and other labs) could prompt early lifestyle or pharmacological intervention, improving patient outcomes at both the individual and population level. Of course, some of the key features identified in this study are more feasible from a data collection perspective, so the ultimate utility of these types of tools will depend on practice cost-benefit analyses in real-world contexts.

## 5 Conclusion

This study proposed an XGB framework with LASSO feature selection as a ML model for predicting the incidence of MHT in a young, apparently healthy population from a LMIC. The proposed model achieved a higher ROC AUC and demonstrated higher scoring metrics compared to the current “rule-of-thumb” classifier for MHT. SHAP analysis found that the office measurements of blood pressure, body weight, DHEA-S biochemical levels, and LV mass were the most important predictors of MHT. PDP revealed the relationships between each LASSO selected feature and the prediction of MHT. Overall, this study demonstrated the promise of using LASSO with an XGB framework model to detect MHT and further development of the model could potentially lead to a viable tool for aiding clinicians in identifying which patients are most at risk for MHT.

## Data Availability

The data analyzed in this study is subject to the following licenses/restrictions: Data sharing is restricted to the terms of the African-PREDICT collaborator network. Requests to access these datasets should be directed to CM, Carina.CM@nwu.ac.za.
