# Supplementary material for: Machine learning model for detecting masked hypertension in young adults
Source: Front Physiol. 2025 Nov 17;16:1684693. doi: 10.3389/fphys.2025.1684693 (PMC12665523; doi:10.3389/fphys.2025.1684693)
Supplement: Supplementary file 1 [file Presentation1.pptx]

## Slide 1
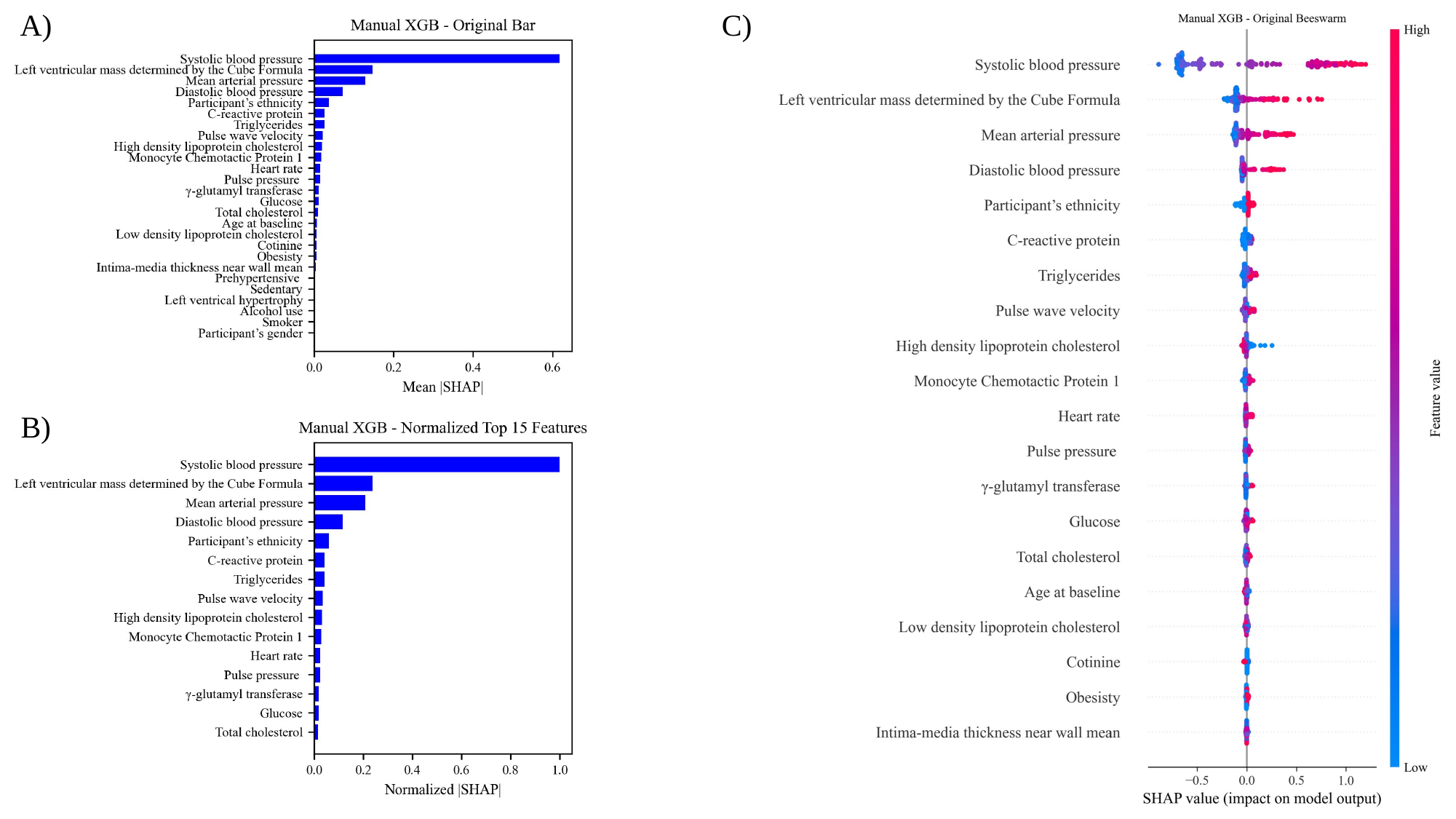

A)
C)
B)

## Slide 2
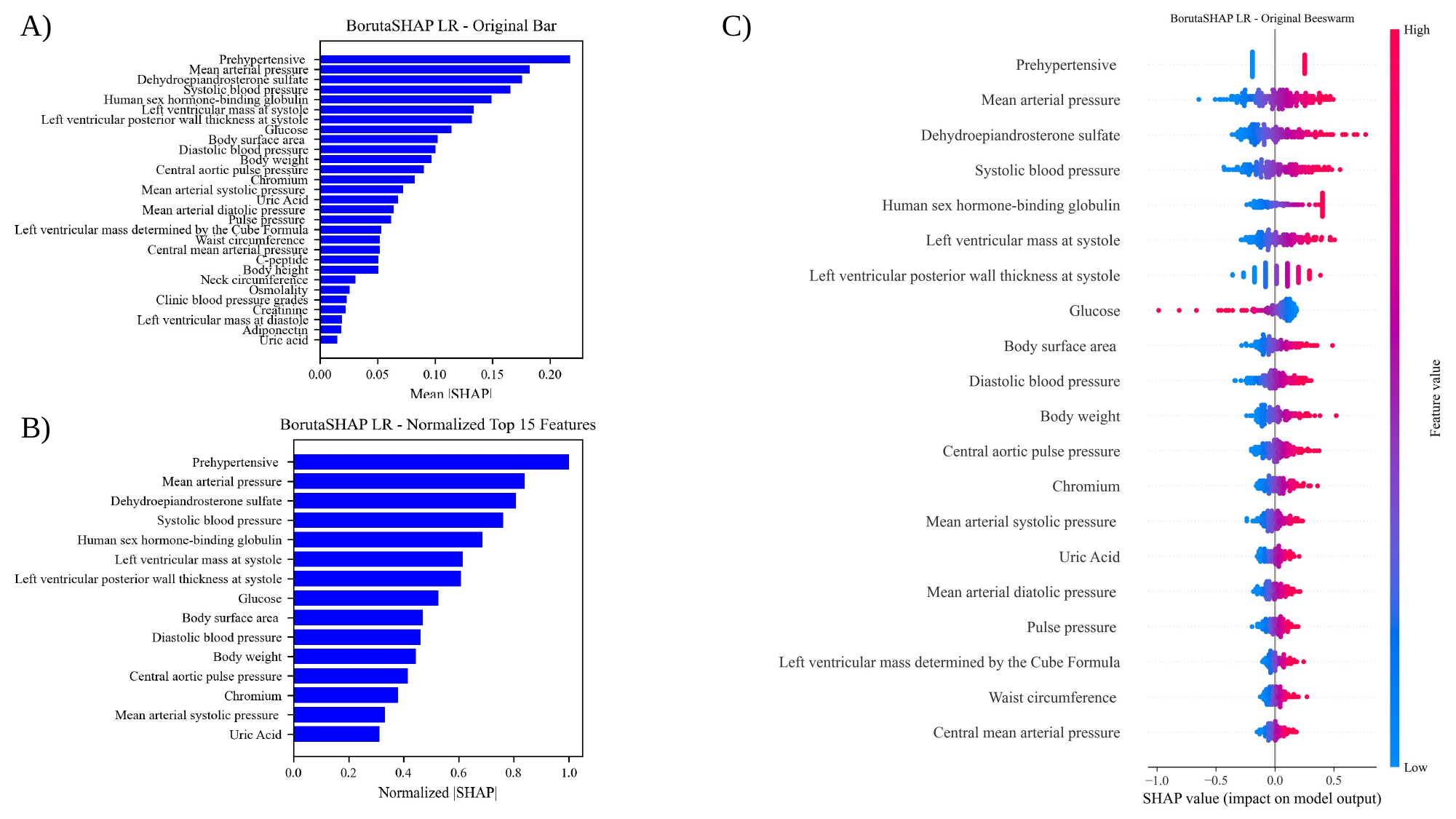

A)
C)
B)

## Slide 3
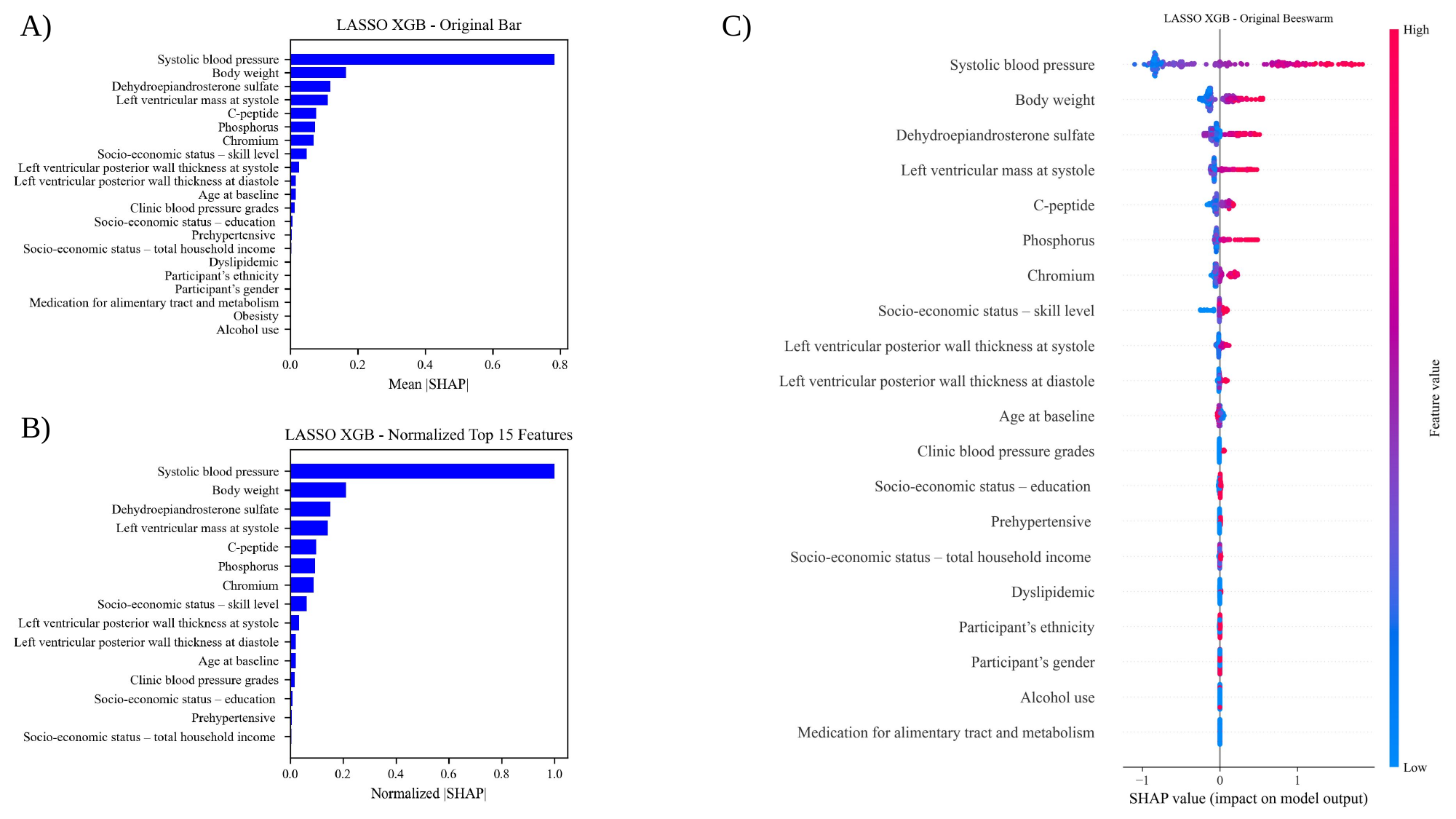

A)
C)
B)

## Slide 4
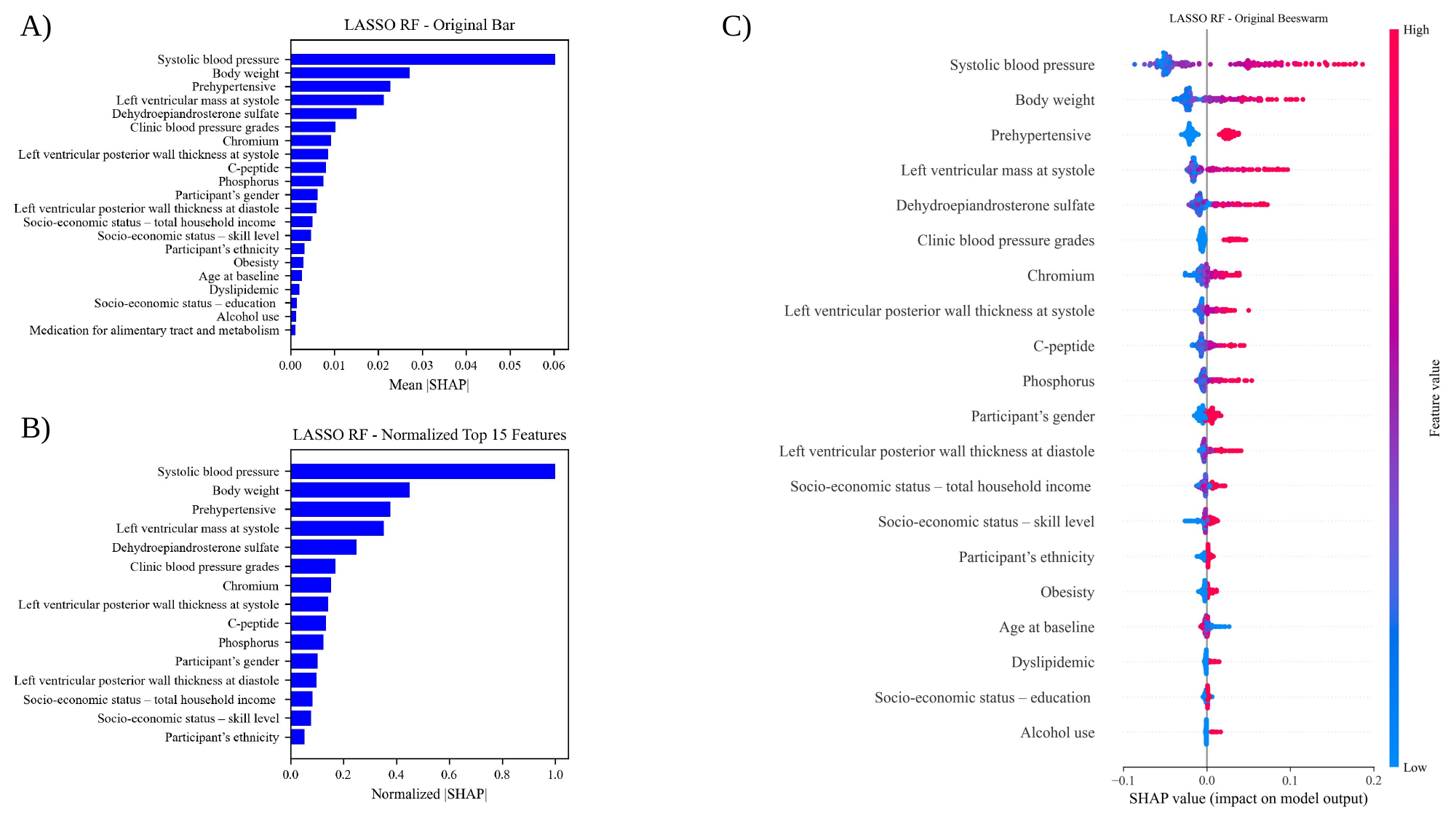

A)
C)
B)

## Slide 5
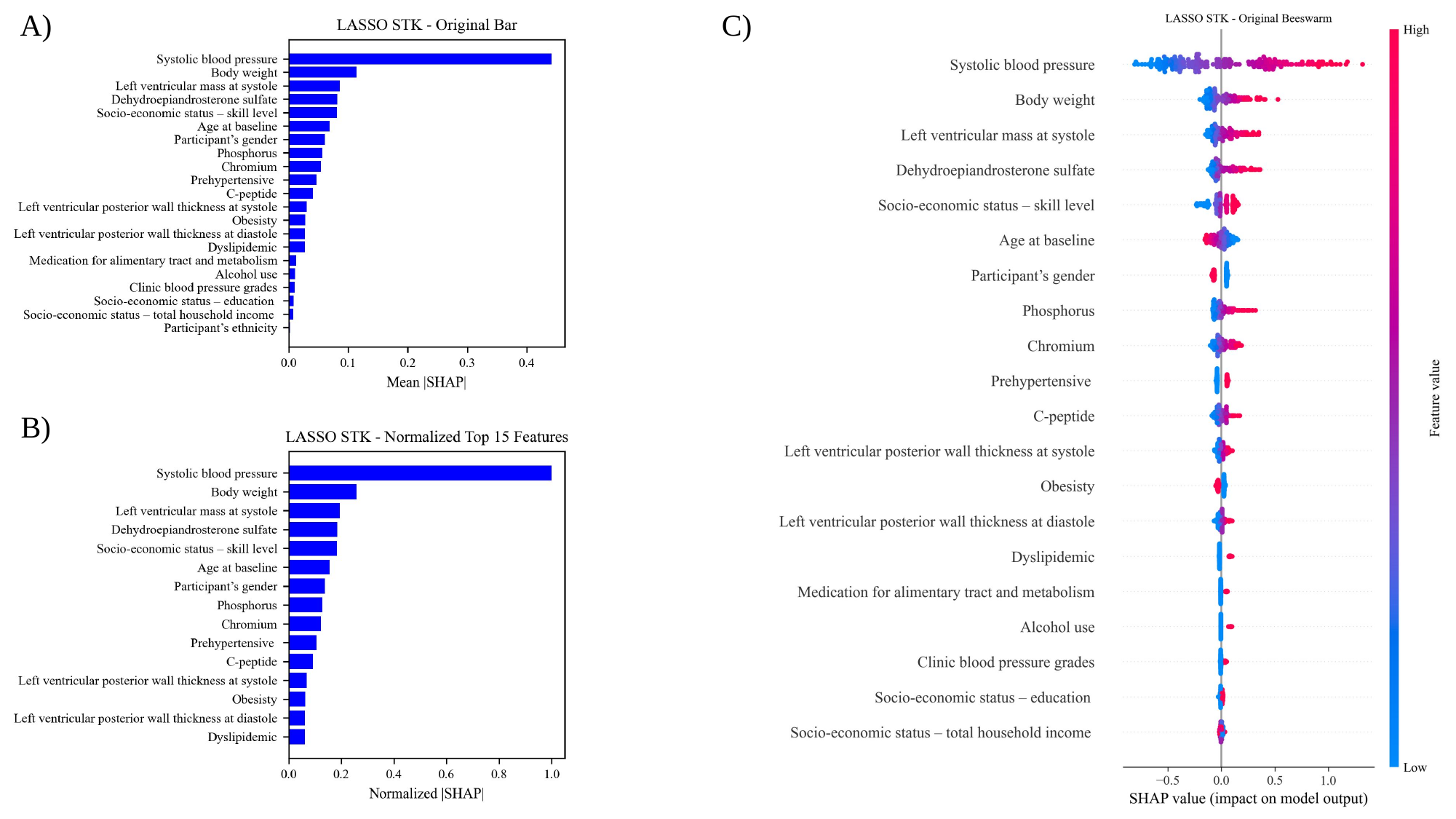

A)
C)
B)

## Slide 6
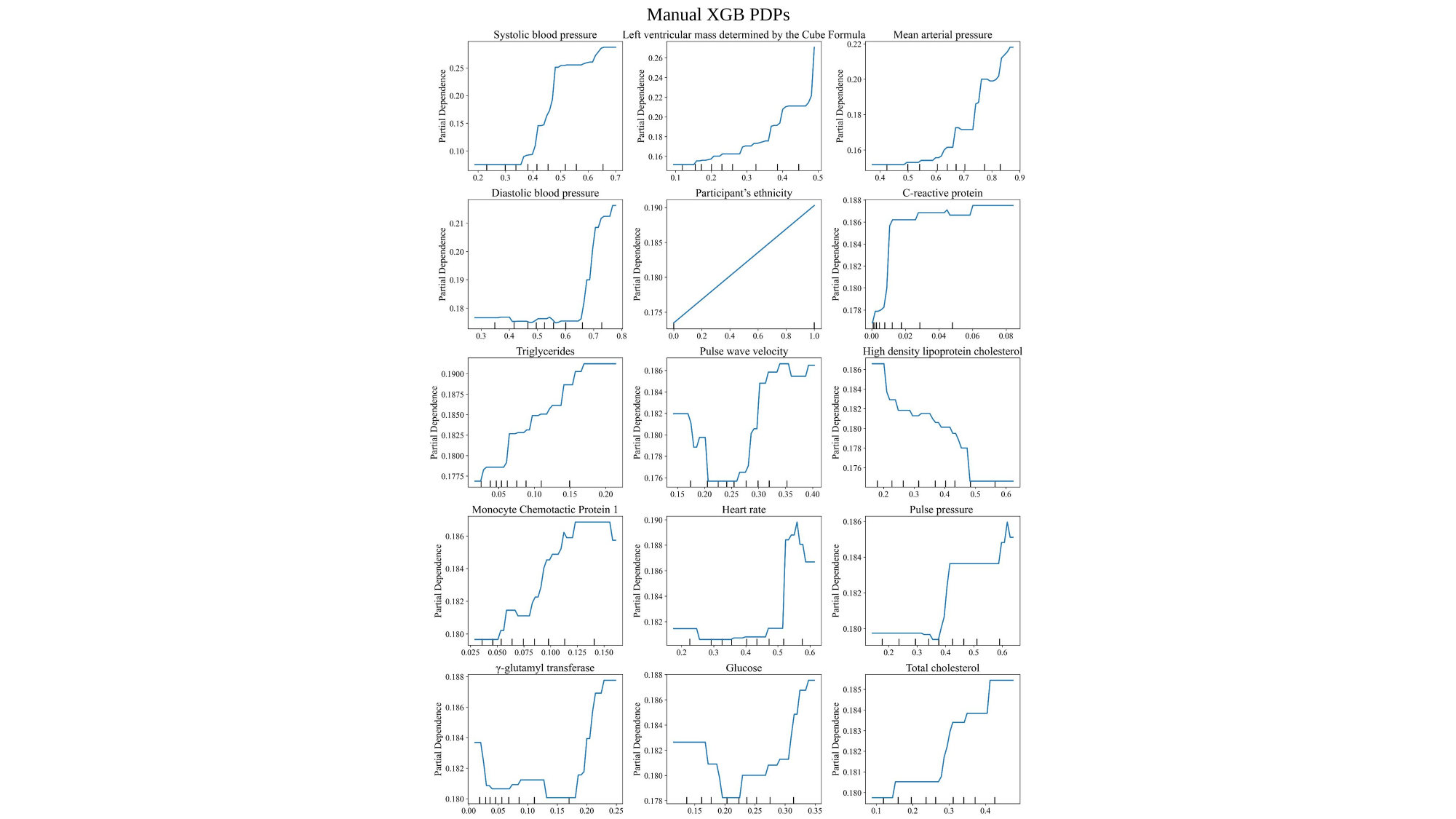

# Manual XGB PDPs

## Slide 7
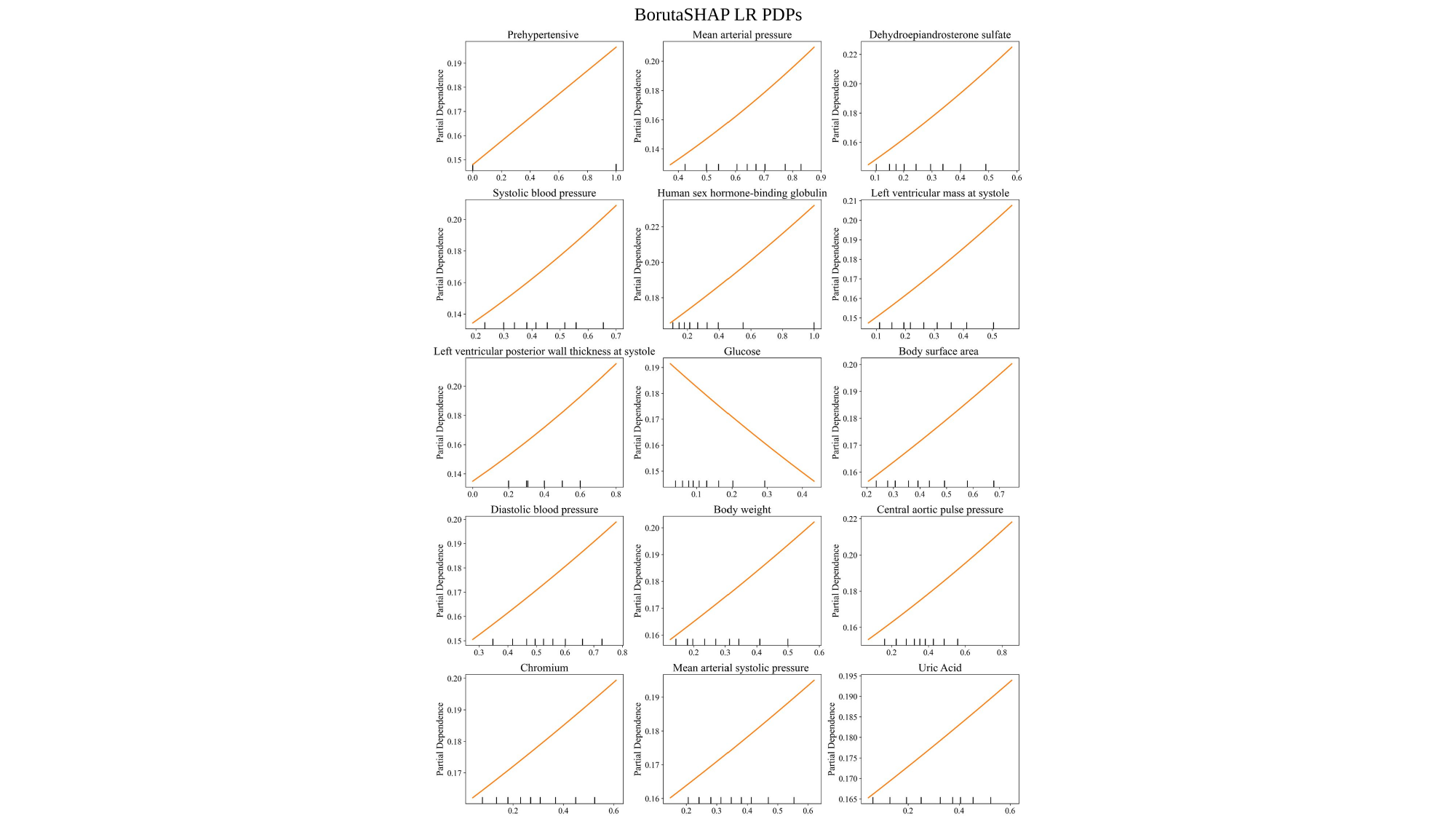

# BorutaSHAP LR PDPs

## Slide 8
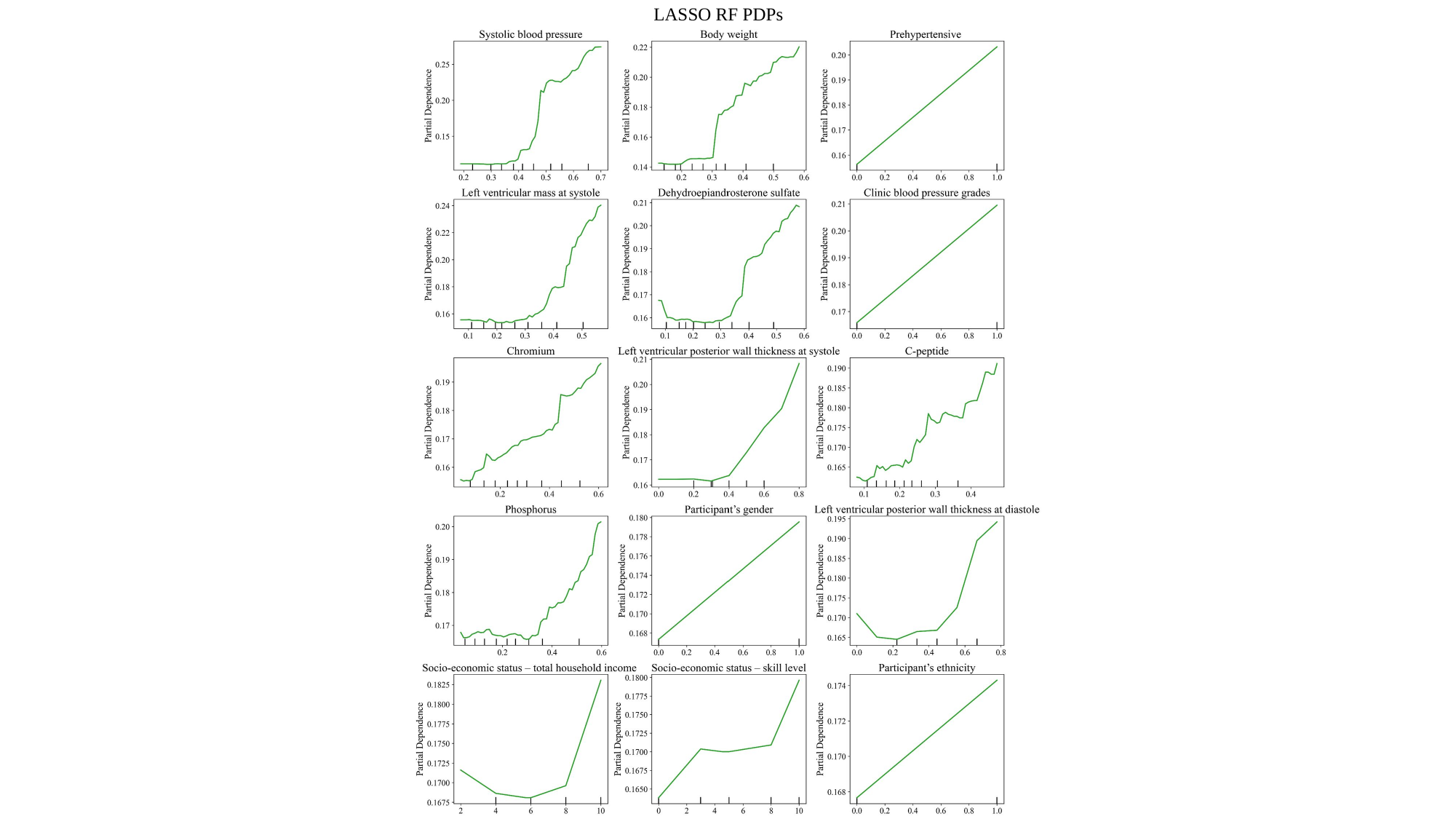

# LASSO RF PDPs

## Slide 9
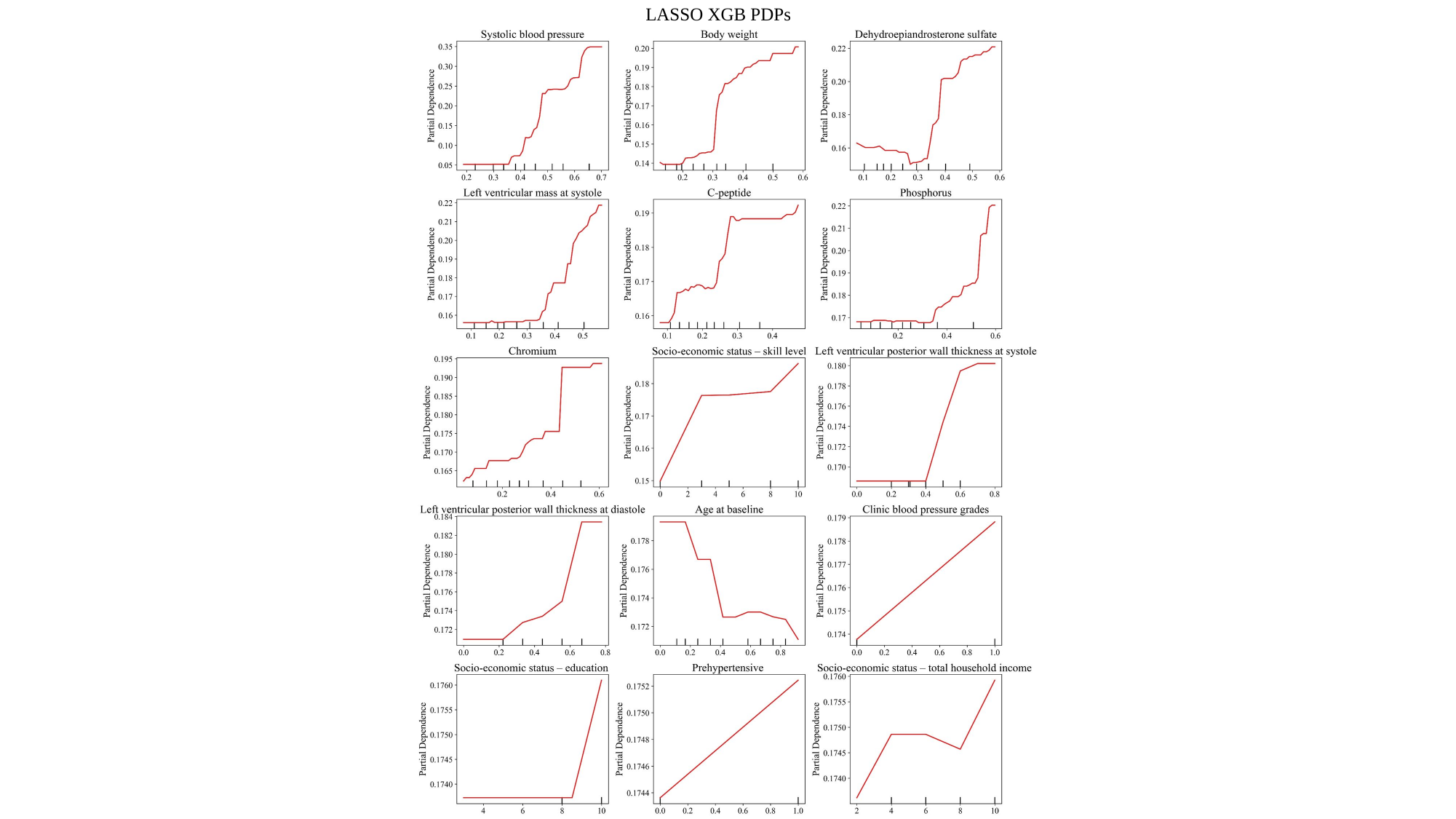

# LASSO XGB PDPs

## Slide 10
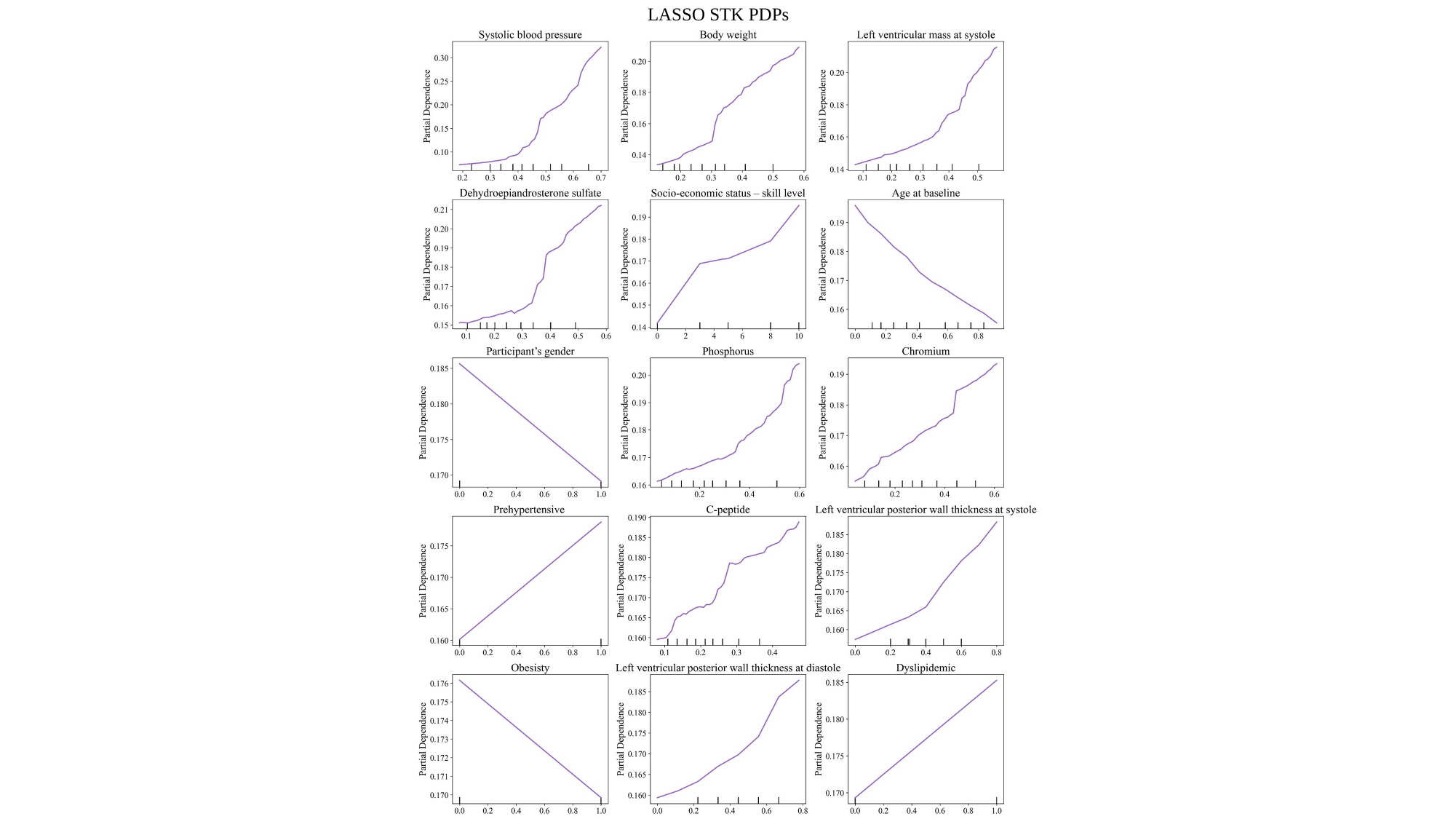

# LASSO STK PDPs
